# Production of transgenic strawberries by temporary immersion bioreactor system and verification by TAIL-PCR

**DOI:** 10.1186/1472-6750-7-11

**Published:** 2007-02-19

**Authors:** Kati J Hanhineva, Sirpa O Kärenlampi

**Affiliations:** 1 University of Kuopio, Department of Biosciences, P.O. Box 1627, 70211 Kuopio, Finland

## Abstract

**Background:**

Strawberry (*Fragaria *× *ananassa*) is an economically important soft fruit crop with polyploid genome which complicates the breeding of new cultivars. For certain traits, genetic engineering offers a potential alternative to traditional breeding. However, many strawberry varieties are quite recalcitrant for *Agrobacterium*-mediated transformation, and a method allowing easy handling of large amounts of starting material is needed. Also the genotyping of putative transformants is challenging since the isolation of DNA for Southern analysis is difficult due to the high amount of phenolic compounds and polysaccharides that complicate efficient extraction of digestable DNA. There is thus a need to apply a screening method that is sensitive and unambiguous in identifying the different transformation events.

**Results:**

Hygromycin-resistant strawberries were developed in temporary immersion bioreactors by *Agrobacterium*-mediated gene transfer. Putative transformants were screened by TAIL-PCR to verify T-DNA integration and to distinguish between the individual transformation events. Several different types of border sequence arrangements were detected.

**Conclusion:**

This study demonstrates that temporary immersion bioreactor system suits well for the regeneration of transgenic strawberry plants as a labour-efficient technique. Small amount of DNA required by TAIL-PCR is easily recovered even from a small transformant, which allows rapid verification of T-DNA integration and detection of separate gene transfer events. These techniques combined clearly facilitate the generation of transgenic strawberries but should be applicable to other plants as well.

## Background

Strawberry (*Fragaria *× *ananassa*) is among the most lucrative agricultural crops worldwide and its consumption has doubled since 1980 [1]. The fruits are rich in bioactive phytochemicals, especially phenolic compounds with high antioxidant capacity, and as a part of daily diet could be beneficial for human health [2]. The conventional breeding programmes aiming at the combination of optimal composition of natural products with outstanding cultivation characteristics and disease resistance are facing a significant barrier caused by the octoploid genome of cultivated strawberry. Breeding through genetic engineering offers a more straightforward strategy by allowing direct introduction of dominant traits to the parent cultivar. Once stably integrated into strawberry genome, the transgene remains in the subsequent rounds of vegetative propagation through runners. The transgene is thus not lost from the complex genome, as might be the case in sexual propagation. Since the first report on the *in vitro *regeneration of strawberry, resulting in the large-scale commercial micropropagation of a crop plant for the first time [3], ample number of protocols for genetic engineering and *in vitro *techniques on strawberry has been developed. During the past few years several reports on improving transgenic strawberry production methods have been published [4-6], and plants e.g. with better fruit firmness [7], freezing tolerance [8] and enhanced resistance to gray mold fungus [9,10] have been achieved. All the reported procedures are usually fine-tuned for certain varieties. There is an apparent need for a widely applicable method that would be suitable for several different cultivars adapted for commercial strawberry production in different climatic conditions.

Bioreactors have become an option for plant *in vitro *multiplication and have been applied for the production of several agricultural and forestry species [11]. A widely used technique is the temporary immersion bioreactor (TIB) where the liquid medium is applied in intervals to the plant material which is located on a separate compartment apart from the medium. The advantages of the TIB systems have been well documented, and benefits have been shown both for reducing workload and thus cost, and for better plant performances by allowing a direct contact of the medium throughout the plant material and by renewing the culture atmosphere on each immersion [12]. Application of the TIB system for *in vitro *regeneration through organogenesis or somatic embryogenesis has been described e.g. for coffee [13], pineapple [14], tea [15], banana [16] and apple [17]. However, only one report has been published where this system was used as a part of regeneration process after *Agrobacterium *mediated callus transformation [18].

In all genetic modification experiments the first important step in the characterization of the putative transformants is to verify the integration of the introduced gene fragment. This is usually achieved by Southern analysis which, however, requires a significant amount of DNA and involves several time-consuming steps [19]. Extraction of pure DNA from sources such as strawberry, which contain high amounts of phenolic compounds and polysaccharides is challenging, as the compounds tend to attach to DNA during purification and interfere with the subsequent enzymatic reactions [20,21].

An alternative approach for the initial screening of transgenic events is to characterize the genomic DNA regions flanking the T-DNA insertion sites. Thermal asymmetric interlaced PCR (TAIL-PCR), a method originally described by Liu and Whittier in 1995 [22,23], is well applicable for this purpose. The strategy is to use nested, T-DNA border region specific primers together with a shorter arbitrary degenerate primer for the unknown genomic DNA region flanking the insertion site. Such priming creates both specific and non-specific products, whose relative amplification efficiencies can be thermally controlled. In three serial PCR reactions the unspecific products are gradually diluted out and in the final reaction the specific products are detectable on the gel by the slight shift in size due to the nested priming on the T-DNA region. Since its development, TAIL-PCR has become an extremely valuable and versatile tool in all research involving recovery of unknown genomic sequences adjacent to known sequences and it has been utilized in functional genomics [24,25], characterization of promoter sequences [26] and also in the detection of genetically modified material in food [27,28]. The application of this technique for the initial screening of transgenic events has not been previously published.

In this report we describe the generation of transgenic strawberries in the TIB system, and the applicability of TAIL-PCR for the detection of individual transgenic events at an early stage of plantlet development.

## Results and discussion

### Regeneration of transgenic strawberries in TIB

By using several strawberry cultivars we have shown previously that the temporary immersion containers are a good environment for the regeneration and organogenesis of strawberry [29]. For the present studies cv. Jonsok was chosen as it is a commonly cultivated Scandinavian variety which has good frost tolerance and firm and aromatic fruits used both as fresh fruits and for processed products [30]. This cultivar could serve as a starting material for biotechnical improvement to develop elite strawberry varieties for northern latitudes.

**Figure 1:**
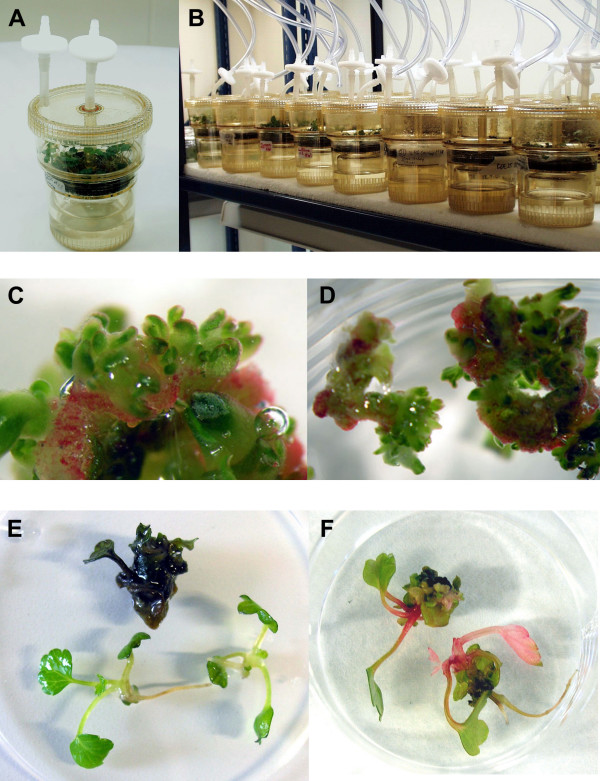
**Organogenesis of transgenic strawberry in Temporary Immersion Bioreactors**. **A**: TIB container (RITA™, Cirad, France) consisting of two compartments. The lower part holds the medium and the plant material is located on a polysulfone foam disc in the upper part of the bioreactor. **B**: Bioreactors in cultivation room connected to automated air pressure system. The overpressure is applied to the lower compartment through sterile filter, and the air pushes the medium to the upper part immersing the plant material. The pressure is released through another filter in the lid of the container, simultaneously renewing the atmosphere in the container. **C **and **D**: Regenerating leaf tissue from strawberry cv. Jonsok in MS medium supplemented with TDZ (2.0 mg/l) and IBA (0.5 mg/l). Regeneration occurs via short callus phase, which partly shows as red colouring of the tissue. At this stage the pieces are transferred into the selection medium. **E**: Strawberry plantlets developed in TIB selection with hygromycin (15 mg/l). Putative transgenic shoots remain green and the rest of the plant material is necrotic. **F**: Plantlets regenerating in kanamycin (50 mg/l). The plantlets are partly bleached and partly green, not giving clear indications of successful gene transfer.

The *Agrobacterium*-mediated gene transfer protocol was started with standard co-cultivation of strawberry on semi-solid medium, followed by transfer to the TIB containers for regeneration (Fig. 1A and 1B). The leaf pieces were placed in the container with 200 ml of medium, at the immersion frequency of ten seconds every four hours, and the medium was changed at two to three week intervals. The leaf pieces started to regenerate three weeks after the onset of the experiment, on average, and gradually produced new shoots as long as they were kept in the regeneration medium, which was usually eight weeks. Strawberry regenerates in this medium as dense clusters of emerging shoots after a short callus phase. When tightly folded young leaves are used as starting material, the leaf pieces are often completely covered with regenerating plant material (Fig. 1C and 1D). At the stage where regeneration started and adventitious buds appeared, the pieces were transferred into another container, and the regeneration medium was supplemented with the selective antibiotic.

### Antibiotic regime in the TIB system

**Figure 2:**
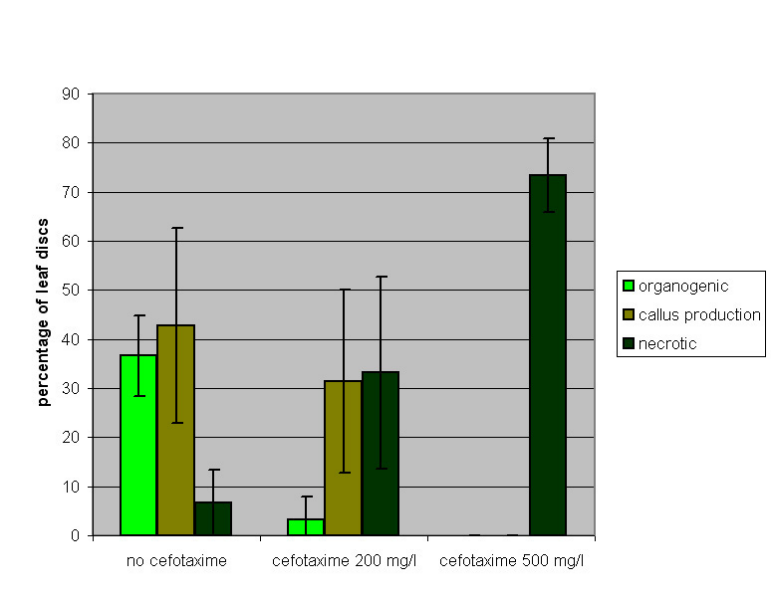
**Effect of cefotaxime on strawberry leaf tissue regeneration**. Leaf discs were cultivated on semi-solid regeneration medium for eight weeks with two-week subculturing intervals. The percentage of leaf discs regenerating, producing only callus or showing total necrosis was calculated. The number of leaf discs on each agar plate was 20–50, and the number of replicate plates for each treatment was 3–5. The error bars show the standard deviation of the percentages of the discs for each treatment.

**Figure 3:**
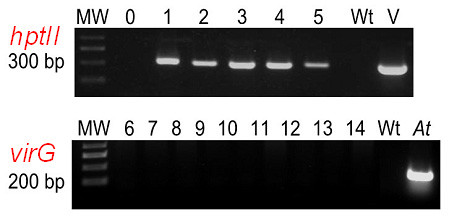
**PCR by *hptII*- and *VirG*-specific primers**. Putative transformants were screened by PCR. The presence of T-DNA was verified by *hptII*-specific primers and the possible *Agrobacterium *contamination was screened by *Agrobacterium virG*-gene specific primers. MW, molecular weight marker; 0, PCR blank; 1–14, putative transgenic strawberry DNAs; Wt, wild-type strawberry DNA; V, pCAMBIA1391Z vector; *At*, *Agrobacterium *DNA.

In *Agrobacterium*-mediated transformation, control of bacterial overgrowth with antibiotic is necessary, and total elimination of A*grobacterium *from the regenerating tissues is inevitable in order to avoid false positive signals in PCR. Cefotaxime is the most effective antibiotic for controlling *Agrobacterium *strain LBA4404 [31]. On the other hand, 100 mg/l of cefotaxime has inhibitory effect on the regeneration of shoots from leaf discs of the diploid strawberry species *Fragaria vesca *and *F. vesca semperflorens *[31]. Also in our studies, cefotaxime showed inhibitory effects on the regeneration of cv. Jonsok, when tested on agar-based regeneration medium. At 200 mg/l, cefotaxime reduced the regeneration significantly and at 500 mg/l it caused severe necrosis and death of the leaf tissue (Fig. 2). However, as the use of liquid medium facilitates an effective and rapid removal of the bacterium without the need of prolonged exposure, a concentration of 200 mg/l of cefotaxime was applied for two weeks in the present study. This short exposure did not inhibit the regeneration process but eliminated the bacterium effectively, as confirmed by the lack of amplification with *VirG *gene-specific primers (Fig. 3).

The most commonly used selectable marker genes in the genetic transformation of plants are *npt*II (neomycin 3'-*O*-phosphotransferase) and *hpt *(hygromycin phophotransferase), which confer resistance to kanamycin and hygromycin, respectively. Both antibiotics have been used in strawberry transformation, and a variety of concentrations have been shown to be effective depending on the cultivar; kanamycin has been used frequently at the concentrations of 50–70 mg/l [10,32-35] and 25 mg/l [5,7,9]. However, Houde *et al*. [8] used up to 450 mg/l of kanamycin for cv. Chambly, while as low as 5 mg/l was effective for selecting transformants in a new cultivar LF9 [6]. In the case of hygromycin, a concentration of 10 mg/l has been applied [33,36]. Also higher sensitivities to hygromycin have been reported, as 1,5 mg/l was appropriate for cv. LF9 [6], and 4 mg/l for the diploid *Fragaria vesca *[37]. Based on these studies, commonly used concentrations of kanamycin (50 mg/l) and hygromycin (10 mg/l) were chosen for the TIB system. In the liquid culture conditions, kanamycin incurred only partial bleaching of the emerging shoots, still allowing growth and making it difficult to distinguish between the transformed and non-transformed shoots. On the other hand, hygromycin was effective, causing clearly distinguishable, severe tissue necrosis (Fig. 1E and 1F). As the impact of hygromycin was thus easier to monitor visually, it was chosen for the subsequent transformation experiments.

The inhibitory action of antibiotics on the onset of regeneration is well known, and for hygromycin it has clearly been demonstrated in soybean [38]. No shoot formation from *Agrobacterium*-treated soybean tissue was observed, when the selection pressure was applied directly after co-cultivation. Delaying the selection by 14 days allowed the generation of transformed shoots. We have a very similar experience with strawberry cv. Jonsok based on several gene transfer experiments in which regeneration failed if the regeneration process was not induced either on semi-solid cultivation or in the TIB system before the direct contact to the antibiotic. In the present study, antibiotic selection was started only after the emerging buds were visible (gradually two to eight weeks). At this stage the leaf pieces were picked into another container and the regeneration medium was supplemented with 10 mg/l of hygromycin. The selective effect was visible within two weeks, the non-transformed senesced plant material being clearly distinguishable from the green and vital, putatively transformed shoots (Fig. 1E).

In order to eliminate possible chimeras and non-transgenic escapes from transgene tests, an iterative selection phase with gradually increasing concentration of the selective agent has been claimed as essential for accomplishing pure transgenic strawberry lines. Since first described by Mathews et al. [39], the iterative selection method has been applied for transgenic strawberry production also by other researchers [8,10,31]. In our study, one more renewal of the regeneration medium was done after selecting the transformants with 10 mg/l hygromycin, and the concentration of hygromycin was increased to 15 mg/l. With this regime, the putative transformants remained viable and the best shoots started to initiate roots (Fig. 1E). The number of transgenic regenerants from one TIB container with the leaf mass from two to three folded leaves varied from two to ten. In this experiment, five tightly folded young leaflets were used as starting material, and altogether 26 regenerants were transferred to soil. Of the regenerants, 21 were transgenic based on *hptII*-PCR, which indicates that the selection with 10 mg/l hygromycin followed by 15 mg/l hygromycin was effective in distinguishing the transgenic plantlets from the non-transformed ones, although even a higher hygromycin concentration in the second selection round might further decrease the number of non-transgenic escapes. After the selection phase, the plantlets were left in the TIB containers with MS medium until shoot formation was vigorous and root formation visible, followed by the acclimatization and planting to soil. There is no evidence of chimerism after several rounds of runner propagation, as all the vegetatively propagated runner plantlets have been transgenic based on PCR screening.

Application of the medium in liquid form brings the active components into effective contact with the treated plant material. A close contact with antibiotics promotes effective selection, which has been reported in ryegrass with hygromycin [40]. In that study, the recovery efficiency of transformed hygromycin-resistant clones was higher when the antibiotic was applied in liquid medium (38% of dishes producing transgenic calli) rather than in semi-solid medium (14% of dishes producing transgenic calli).

### DNA purification from strawberry leaves

A wealth of reports are available for the extraction of DNA from strawberry tissues, but none of them has proven to be applicable for all cultivars, and no single practice among different laboratories exists [20,21,41,42]. Gaining pure DNA from strawberry is difficult because of the high content of interfering phenolic compounds and polysaccharides. Pre-treatment with polyvinylpyrrolidone (PVP) or polyvinylpolypyrrolidone (PVPP) before DNA precipitation to bind the phenolic components before DNA precipitation has been suggested as a helpful step in the purification process [20,21,42] but to our experience the recovered DNA may not be sufficiently pure for enzymatic reactions. The main factor affecting the quality of DNA obtained from strawberry leaf, which is the part in the developing plantlet normally used for DNA extraction, is the age of the leaf. We have used a commercial DNA extraction kit for purifying DNA from mature, dark green leaves and from young, pale green folded leaves. When a maximum amount of starting material (100 mg) is loaded in the kit, DNA with the A_260_/A_280 _absorbance ratio of 1.8, characteristic for pure DNA without contaminating proteins, and A_260_/A_230 _value above 1.8, illustrating the absence of phenolic compounds, can be routinely achieved from the young leaves. However, when the same amount of dark green mature leaves is used, the typical value of A_260_/A_280 _is 1.5 and the value of A_260_/A_230 _is below 1, indicating the presence of contaminating compounds. Also the total yield of DNA from young tissue is higher (3–5 μg of DNA/100 mg young leaf vs. 1–2 μg of DNA/100 mg mature leaf material, data not shown), as the DNA content is higher in young leaves due to a smaller cell size. Thus, based on our experience on cv. Jonsok, the main difficulties in DNA extraction can be overcome by the careful choice of starting material. The most suitable plant part is the folded young leaf, since it has lower amount of interfering compounds and higher content of DNA compared to the mature leaf. Furthermore, purification of DNA with a kit is rapid, and the small amount of leaf material (20 mg) needed is easily obtained from a very young plantlet. Pure DNA can be recovered when the final elution volume of the kit is adjusted to the amount of starting material.

### Analysis of T-DNA integration

Screening of positive transformants by Southern analysis is time-consuming and involves several steps, including extraction of large amount of pure DNA. In order to get enough DNA (10–20 μg) for Southern analysis from one putative transformant, either several emerging leaves need to be collected or the plant needs to be multiplied via runner proliferation. Both ways are time-consuming and the genotyping of a set of transgenic strawberries may take the entire first growing season. Nevertheless, a careful monitoring of each individual is inevitable to distinguish between the different transgenic events, because the regeneration of strawberry typically occurs from tight clusters of emerging shoots, which can originate from the same *Agrobacterium*-transformed cell. With a technique that involves cloning of the DNA, a small amount (10–100 ng) of DNA is sufficient and easily achievable already from a very small plantlet by commercial plant DNA extraction kits. Once PCR-grade pure DNA sample is extracted, it serves as a template for preliminary screening of positive transformants, and can subsequently be used for TAIL-PCR analysis to verify independent transformants.

**Figure 4:**
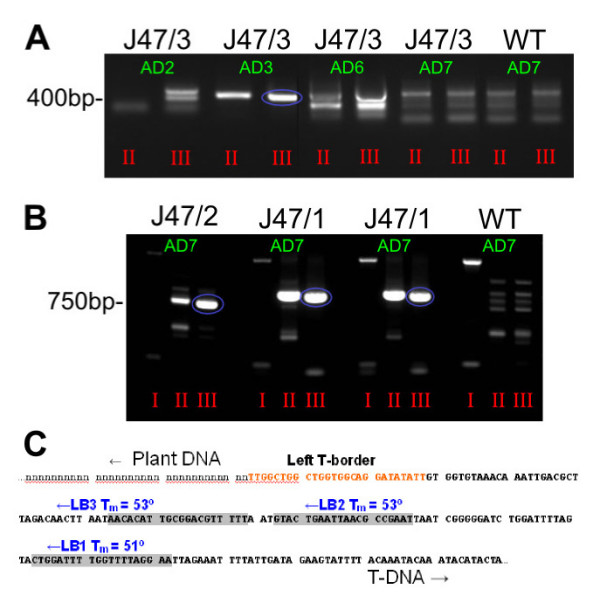
**Agarose gel electrophoresis of TAIL-PCR products**. **A: **TAIL-PCR (secondary and tertiary reactions are shown) from one putative transgenic line with four different arbitrary primers together with wild type control with one arbitrary primer. Only AD3 primer produced specific product recognizable from the slight shift in size due to the nested LB priming at the T-DNA junction site. Other arbitrary primers did not produce clear, specific products; only faint non-specific bands also present in the wild type control are seen, as exemplified by AD7 primer. **B: **TAIL-PCR reactions with the same arbitrary primer (AD7) on three putative transgenic plants and a wild type control, all three TAIL-PCR reactions shown. The primary TAIL-PCR reaction produces only small amount of the specific product, not yet visible on the gel, and the specific products start to emerge at the secondary reactions. Two of the three samples shown here proved to originate from the same gene transfer event (J47/1), and one showed a different product pattern, thus being a different event (J47/2). No specific products are shown in the wild type control. Strawberry clones: J47/1–3; WT, wild type. Arbitrary primers: AD2, AD3, AD6, AD7. TAIL-PCR reactions: I, primary (LB1 primer); II, secondary (LB2 primer); III, tertiary (LB3 primer). Specific TAIL-PCR reaction products excised from gel for sequencing are marked with blue oval. **C**: Position of T-DNA junction site specific primers at the left T-DNA border region of the pCAMBIA 1391Z gene transfer vector. The three nested T-DNA left border specific primers were designed for the TAIL-PCR analysis. The vector sequence is shown on uppercase and the left T-DNA border region as orange colour.

**Figure 5:**
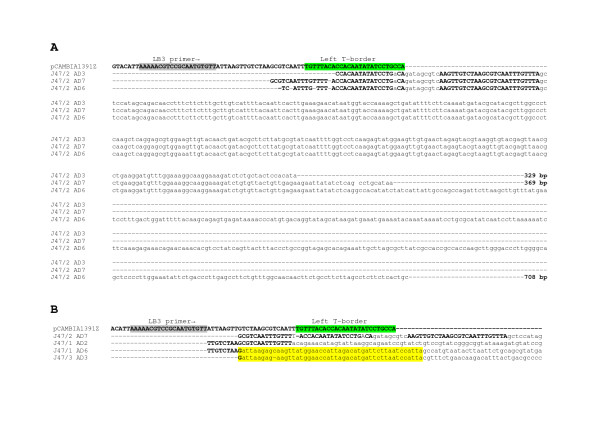
**Sequence alignments of left T-DNA junction sequences of transgenic strawberries**. **A**: Sequence alignment of the same insertion site with three different arbitrary primers. Transgenic strawberry clone J47/2 produced specific TAIL-PCR products with three different AD primers. Sequences of these products showed one T-DNA junction site, which was primed at three different positions. Sequence alignment of the three fragments is shown together with the left T-border region of the pCAMBIA 1391Z gene transfer vector shown on the first line. Sequence homologous to pCAMBIA 1391Z is shown on bold uppercase. Duplication of 26 bp of the T-DNA border region is seen after 12 bp filler DNA fragment of unknown origin. **B**: Sequence alignment of the left T-DNA border DNA at different insertion sites. The three different strawberry clones showed different arrangements at the left T-DNA border regions. Two separate insertion sites from one clone (J47/1 AD2 and J47/1 AD 6) are shown. Sequence homologous to pCAMBIA 1391Z is marked bold uppercase. A homologous segment of unknown origin found in two border regions is marked yellow.

Strawberry genomic DNA flanking the inserted T-DNA left border was cloned by TAIL-PCR using three nested T-DNA-specific primers (Table 1, Fig. 4) together with four arbitrary degenerate primers (Table 1). The products from the three TAIL-reactions were separated on agarose gel, and the specific products were identified on the basis of size shift and absence of similar products from the wild type sample (Fig. 4). The specific bands were cut out from the gel and sequenced. All of the ten individual plants characterized gave specific TAIL-PCR products with at least one of the four arbitrary primers, ranging in size between 300–750 bp. When the products were sequenced, only three different sets were found, which suggests that several transgenic shoots have emerged from the same callus cluster originating from a single transformed cell. For one of the three lines (J47/2), as many as three arbitrary primers gave specific products and, when sequenced, showed a single T-DNA insertion primed by three arbitrary primers, proving well the suitability of this set of arbitrary primers for strawberry genomic DNA (Fig. 5A). For another transformant, two different sequences were obtained from the reactions with two different arbitrary primers, suggesting two separate insertion sites (Fig. 5B).

Rearrangements, such as duplication or translocation of the target genome sequence and insertion of filler DNA, are commonly observed in the T-DNA junction site as a result of illegitimate recombination during the integration process [43-45]. In the T-DNA/strawberry DNA junction such rearrangements were also seen. Sequencing of three different T-DNA junction sites all indicated different arrangements (Fig. 5B) showing the imprecise and poorly conserved junction typical particularly for the left border insertion site of T-DNA [43,46]. A short segment of vector DNA from the T-DNA left border region was usually present (Fig. 5). A 26 bp duplication of the T-DNA border region was detected in one of the transformants, with a 12 bp filler DNA fragment of unknown origin (Fig. 5B).

With TAIL-PCR, already the different amplification patterns seen on agarose gel provide information about the different transgenic lines, enabling one to confirm the presence of separate transformation events already within a few days from the first DNA extraction of the shoots. Whenever the junction sites need to be further characterized, the cloned T-DNA-flanking DNAs can be extracted from the gel and sequenced. Compared to Southern analysis, this method does not give conclusive information about the number of insertions in the individual tranformants. TAIL-PCR may produce several sequences and indicate several insertion sites, but there may still be some insertion sites that do not give products in TAIL-PCR. For accurate copy number, Southern analysis is still a proper method of choice.

For the safety assessment of genetically modified crop plants, sequencing of the transgene junction sites may be required [47,48]. TAIL-PCR provides a convenient way to generate these data and gives a good basis for the molecular level selection of the "cleanest" transformation events for further commercial development. Further applications of TAIL-PCR in strawberry research include T-DNA mutagenesis research for both reverse and forward genetics purposes. Such studies are ongoing with the diploid strawberry *Fragaria vesca *[37] and the results shown in this report by octoploid *Fragaria *× *ananassa *suggest that TAIL-PCR could be used for the diploid relative as well. Overall, the method can be utilized for resolving the structure of genes in the Rosaceae plant family.

## Conclusion

Development of transgenic plants is an indispensable tool both in functional genomics research and also in modern plant breeding. For many plant species, protocols have been developed to generate transformants, some plants being more amenable for transformation and regeneration than others. The TIB system proved to be a suitable environment for the generation of transgenic strawberries and provides, as a labour effective method, an easy and convenient system presumably applicable for other plants as well. While in our case it proved to function for a recalcitrant plant, it can reduce workload also in the case of routine gene transfers by allowing more convenient handling of plant material.

Verification of the presence of transgene in the plant genome and distinguishing between the independent transformation events can be time-consuming, as usually large amount of DNA is needed to accomplish the analysis. A method that involves cloning of the target sequence enables the analysis from a smaller amount of plant material. With TAIL-PCR, distinguishing between different transgenic lines is possible already from the first plantlet. With the combination of TIB and TAIL-PCR, an effective system can be set up for the generation and characterisation of transformed plants, exemplified here by strawberry.

## Methods

### Development of genetically modified strawberries

Plant gene transfer vector pCAMBIA1391Z containing the hygromycin selectable marker gene was used for the gene transfer (Cambia org., Canberra, Australia). The construct was transferred to *Agrobacterium tumefaciens *LBA4404, using a standard freeze-thaw method [49]. Plant material from strawberry cv. Jonsok was sterilized and chopped [29]. The leaf pieces were incubated in *A. tumefaciens*/MS suspension [50] for 30 min, briefly dried on Whatman filter paper and co-cultivated on MS agar plate overnight. For regeneration, the pieces were placed into temporary immersion bioreactor (TIB) containers (RITA™, Cirad, France) together with the regeneration medium, i.e. MS medium supplemented with thidiazuron (TDZ; 2.0 mg/l) and indole butyric acid (IBA; 0.5 mg/l) [29]. Occasional contaminations were controlled by immersing the plant material in MS medium with 10 ml/l plant preservative mixture (PPM™, Plant Cell Technology, Washington, USA) for 30 min and adding PPM to the regeneration medium (2 ml/l). For the first two weeks cefotaxime (200 mg/l) was added in order to eliminate *A. tumefaciens*. The selective antibiotic hygromycin (10 mg/l for the first two to four weeks followed by 15 mg/l for one to two weeks) was applied as the regenerating shoots started to emerge. Shoots that continued developing in the presence of hygromycin were transferred to another container containing MS medium for rooting. The conditions in the cultivation room were: light intensity 45 μmol m^-2 ^s^-1^, duration of light period 19 hours at 22°C. The immersion frequency in the TIB system was 10 s every four hours.

### Testing the effect of cefotaxime on regeneration

Leaf discs were surface-sterilized and cut into small pieces, and cultivated on semi-solid regeneration medium in the cultivation room for eight weeks with subculturing intervals of two weeks. The effect of 200 and 500 mg/l of cefotaxime was tested. The percentage of leaf discs regenerating, producing only callus or showing total necrosis was calculated. The number of leaf discs on each agar plate was 20–50, and the number of replicate plates for each treatment was 3–5.

### T-DNA junction analysis

Genomic DNA from the putative transformants was extracted from young folded leaves with DNeasy^® ^Plant Mini Kit (Qiagen). DNA concentration and purity was analyzed by NanoDrop^® ^ND-1000 Spectrophotometer (NanoDrop Technologies). All PCR reactions were carried out in Go Taq^® ^Green Master Mix (Promega) according to manufacturer's instructions. For conventional PCR, the primer concentration in the reactions was 0,4 pmol/μl and the amount of template DNA was 50–100 ng. The primer sequences are shown in Table 1 and cycling conditions in Table 2. The instrument used for conventional PCR was PTC-100™ Programmable Thermal Controller (MJ Research Inc.).

The T-DNA junction site sequences from the left border were determined by the TAIL-PCR method [22]. The TAIL-cycling was included also for the tertiary PCR reaction, as it was recognised to be necessary to further distinguish between the specific and non-specific products (Table 2). The primer concentrations in the primary and secondary TAIL-PCR reactions were 0,6 pmol/μl and 1,3 pmol/μl for the left border-specific and arbitrary primers, respectively. In the tertiary TAIL reactions the concentrations of both primers were 0,6 pmol/μl. The primary TAIL reactions contained 100–200 ng of genomic DNA. All TAIL-PCR reactions were carried out in iCycler iQ™ (Bio-Rad).

The specific PCR products from the tertiary TAIL-PCR reactions were extracted from agarose gel with QIAquick^® ^Gel Extraction Kit (Qiagen). The DNA fragments were sequenced using Thermo Sequenase CY5 Dye Terminator Kit (Amersham Biosciences) and automated sequencer A.L.F. express DNA sequencer (Amersham Biosciences).

## Authors' contributions

KH designed the experiments, did the laboratory work and wrote the first manuscript. SK is the research group leader and contributed to the experimental design and finalizing the manuscript. All authors have read and approved the final manuscript.

## Figures and Tables

**Table 1:** Primers used for PCR analysis

**PCR experiment**	**name**	**sequence (5'-3')**	**Reference**
Transgene detection	hptIIF	ACGAGCGGGTTCGGCCCATT	this work
	hptIIR	CGGCTCCGGATCGGACGATT	
			
*Agrobacterium *control	virGF	GCCGACAGCACCCAGTTCAC	[51]
	virGR	CCTGCCGTAAGTTTCACCTCACC	
			
TAIL-PCR, T-DNA specific	LB1	TTCCTAAAACCAAAATCCAG	this work
	LB2	ATTCGGCGTTAATTCAGTAC	
	LB3	AAAAACGTCCGCAATGTGTT	
			
TAIL-PCR, arbitrary^a^	AD2	NGTCGASWGANAWGAA	[23]
	AD3	WGTGNAGWANCANAGA	[23]
	AD6	WGCNAGTNAGWANAAG	[52]
	AD7	AWGCANGNCWGANATA	[52]

^a^IUPAC-IUP codes for the wobble bases: W = A or T, N = G or A or T or C

**Table 2:** Thermal conditions in PCR analysis

**PCR with *hptII *and *virG *primers**	**Primary TAIL PCR**	**Secondary and tertiary TAIL PCR**
95°C 3 min	95°C 3 min	95°C 3 min
		
*25 repeats*:	*5 repeats*:	*5 repeats*:
95°C 30 s	95°C 20 s	95°C 20 s
62°C 30 s	51°C 1 min	53°C 1 min
72°C 1 min	72°C 2,5 min	72°C 2,5 min
		
72°C 7 min	*15 repeats*:	*15 repeats*:
	95°C 20 s	95°C 20 s
	51°C 1 min	53°C 1 min
	72°C 2,5 min	72°C 2,5 min
	95°C 20 s	95°C 20 s
	51°C 1 min	53°C 1 min
	72°C 2,5 min	72°C 2,5 min
	95°C 20 s	95°C 20 s
	40°C 1 min	40°C 1 min
	72°C 2,5 min	72°C 2,5 min
		
	72°C 5 min	72°C 5 min
